# Supplementary material for: Pilot Study on Poultry Meat from Antibiotic Free and Conventional Farms: Can Metagenomics Detect Any Difference?
Source: Foods. 2022 Jan 18;11(3):249. doi: 10.3390/foods11030249 (PMC8834493; doi:10.3390/foods11030249)
Supplement: Supplementary file 1 [file foods-11-00249-s001.zip › foods-1535864-supplementary.pdf]

foods

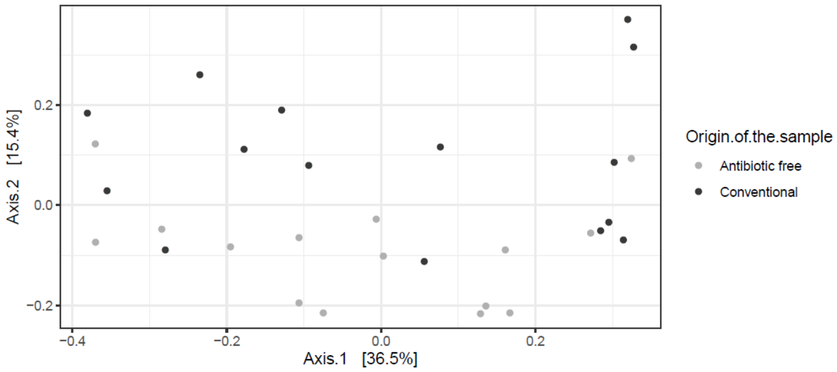

Figure S1. Bray-Curtis dissimilarity plots showing the genera detected on carcasses from the conventional and antibiotic free farm.

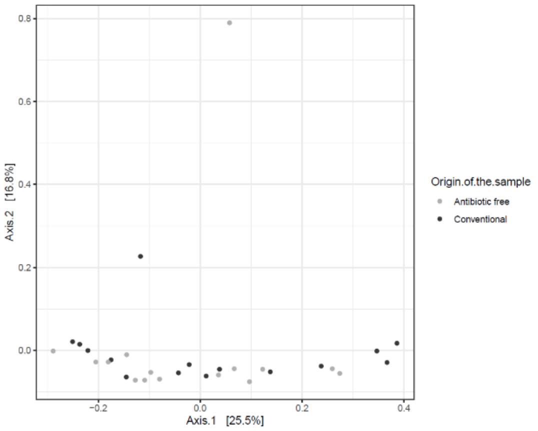

Figure S2. Bray-Curtis plot beta diversity of functional genes in the carcasses of birds reared in the antibiotic free and conventional farm.

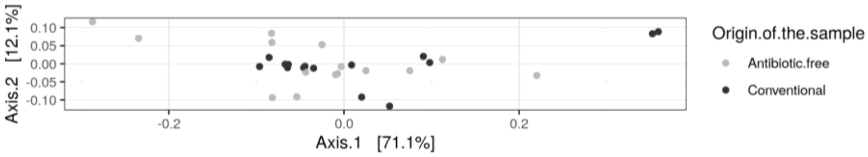

Figure S3. Beta diversity of carcasses samples shown as PCoA of Bray-Curtis diversity computed based on the AMR gene family abundances normalized with DESeq2.

Table S1. Numbers identifying the tested broilers during slaughtering and corresponding metagenome numbers for the caeca and the carcass samples.

Broiler number	Type of farm	Metagenome number for the caeca sample	Metagenome number for the carcass sample
31	Conventional	1343	1280
32	Conventional	1344	1281
33	Conventional	1345	1282
34	Conventional	1346	1283
35	Conventional	1347	1284
36	Conventional	1348	1285
37	Conventional	1349	1286
38	Conventional	1350	1287
39	Conventional	1351	1288
40	Conventional	1352	1289
41	Conventional	1353	1290
42	Conventional	-	1291
43	Conventional	1355	1292
44	Conventional	1356	1293
45	Conventional	1357	1294
46	Antibiotic free	1358	1295
47	Antibiotic free	1359	1296
48	Antibiotic free	1360	1297
49	Antibiotic free	1361	1298
50	Antibiotic free	1362	1299
51	Antibiotic free	1363	1300
52	Antibiotic free	1364	1301
53	Antibiotic free	1365	1302
54	Antibiotic free	1366	1303
55	Antibiotic free	1367	1304
56	Antibiotic free	1368	1305
57	Antibiotic free	1369	1306
58	Antibiotic free	1370	1307
59	Antibiotic free	1371	1308
60	Antibiotic free	1372	1309

Table S2. Normalized mean (DESeq2 normalized counts) of the top 20 genera significantly different ($p<0.05$) in the caeca of the birds reared in the conventional and antibiotic-free farm.

Genus	Norm_mean_ conventional	Norm_mean_ antibiotic free	p-value
Alistipes	121394.483	201764.198	0.00
Alkaliphilus	65854.658	37406.434	0.00
Anaerotruncus	81830.348	141769.053	0.00
Bacillus	121574.408	80982.300	0.00
Bacteroides	903112.032	5541950.681	0.00
Coprococcus	56576.507	94133.578	0.00
Desulfitobacterium	83821.616	49267.150	0.00
Dorea	69937.780	121675.154	0.00

Escherichia	40613.940	215143.602	0.00
Ethanoligenens	126272.735	80004.036	0.00
Holdemania	87562.749	113518.336	0.00
Lactobacillus	162869.829	297992.157	0.02
Parabacteroides	54047.672	557109.972	0.00
Prevotella	41748.154	697359.162	0.00
Roseburia	95006.083	151647.561	0.00
Ruminococcus	522286.960	806960.484	0.00
Streptococcus	122827.116	90076.971	0.01
Subdoligranulum	342496.625	505311.498	0.00

Table S3. Mean values of the InvSimpson, Shannon, Chao1 indexes quantified for the genera identified in the caeca of birds from the conventional and the antibiotic free farm.

	**InvSimpson**	**Shannon**	**Chao1**
Mean_Conventional	13.733	3.747	559.631
Mean_Antibiotic free	7.08	3.067	554.05

Table S4: Norm_Mean (DESeq2 normalized counts) values of the top 20 functional genes significantly different ($p<0.05$) in the caeca of the birds reared in the conventional and antibiotic-free farm.

**Level function**	**Norm_Mean_ conventional**	**Norm_Mean_ antibiotic free**	**p-value**
Beta-galactosidase (EC 3.2.1.23)	7282.341	0	0.00
Chaperone proteinDnaK	9293.178	12738.485	0.00
DNA topoisomerase III (EC 5.99.1.2) in PFGI-1-like cluster	2.005	0.214	0.00
Excinuclease ABC subunit A paralog in greater Bacteroides group	410.247	2013.301	0.00
Integrase	9163.519	15684.380	0.00
Ribonucleotide reductase of class III (anaerobic), large subunit (EC 1.17.4.2)	9822.277	14658.281	0.00
Translation elongation factor G	9751.141	18514.220	0.00

Table S5. Norm_Mean values (DESeq2 normalized counts) of the top 20 genera significantly different ($p<0.05$) in the carcasses of the birds reared in the conventional and antibiotic-free farms.

**Genus**	**Norm_Mean_ Conventional**	**Norm_Mean_ Antibiotic-free**	**p-value**

Aeromonas	118.652	325.106	0.00
Anoxybacillus	702.123	14.224	0.00
Bacillus	1032.502	183.119	0.00
Bacteroides	520.731	2011.121	0.00
Geobacillus	616.229	40.338	0.00
Pedobacter	429.521	127.025	0.00
Prevotella	54.755	339.248	0.00
Ruminococcus	142.057	361.581	0.00
Sphingobacterium	684.452	188.174	0.00

Table S6. Mean values of the InvSimpson, Shannon, Chao1 indexes quantified for the genera identified on carcasses of birds from the conventional and the antibiotic free farm.

Index	Mean conventional	Mean antibiotic-free	p-value
InvSimpson	13.312	10.738	0.44
Shannon	3.233	3.185	0.88
Chao1	491.491	503.355	0.75

Table S7. Genes classified as antibiotic resistant genes in the Level 2 category and showing normalized mean values significantly different ($p<0.05$) in the caeca of birds from the conventional and antibiotic free farm. Genes in bold have been identified also on carcasses but with any statistical significative difference in the two tested groups.

Level function	Norm_Mean Conventional	Norm_Mean Antibiotic free	pval
Level 3-Aminoglycoside_adenylyltransferases			
AminoglycosideN6'-acetyltransferase (EC 2.3.1.82)	8.085	0.743	0.000
**Spectinomycin9-O-adenylyltransferase**	95.174	19.101	0.000
**Streptomycin3''-O-adenylyltransferase (EC 2.7.7.47)**	115.467	324.204	0.000
Level 3- Beta-lactamase			
Beta-lactamase class A	6.453	0.181	0.023
**Beta-lactamase class C and other penicillin binding proteins**	162.999	29.850	0.000
Beta-lactamaserepressorBlaI	395.078	36.980	0.001
Metal-dependent hydrolases of the beta-lactamase superfamily II	16.522	2.465	0.000
**Negative regulator of beta-lactamase expression**	5.177	0.897	0.003
Regulatory protein BlaR1	23.199	0.507	0.000
Level 3- BlaR1_Family_Regulatory_Sensor-transducer_Disambiguation			
Beta-lactamase repressor BlaI	0	51.574	0.000
**Regulatory sensor-transducer. BlaR1/MecR1 family**	1031.434	412.738	0.000

Transcriptional regulator. MecI family	33.457	83.803	0.001
Transcriptional repressor. BlaI/MecI family	261.786	462.161	0.000
Level 3-Erythromycin_resistance			
rRNA adenine N-6-methyltransferase (EC 2.1.1.48)	190.674	712.307	0.000
rRNA adenine N-6-methyltransferase (EC 2.1.1.48) (Macrolide-lincosamide-streptogramin B resistance protein) (Erythromycin resistance protein)	23.962	1.709	0.000
rRNA adenine N-6-methyltransferase (erm/ksgA)	3.797	16.206	0.000
Level 3- Methicillin_resistance_in_Staphylococci			
FmtA protein involved in methicillin resistance	3.757	0.481	0.017
Transposase for insertion sequence-like element IS431mec	0.618	4.946	0.005
**UDP-N-acetylmuramoylalanyl-D-glutamate--2.6-diaminopimelate ligase (EC 6.3.2.13)**	2541.379	0	0.000
Level 3- MexE-MexF-OprN_Multidrug_Efflux_System			
**Multidrug efflux transporter MexF**	67.028	32.709	0.000
**Transcriptional regulator MexT**	1.374	0.113	0.089
Level 3- Multidrug_Resistance_Efflux_Pumps			
**Acriflavin resistance protein**	2292.452	7129.559	0.000
**Macrolide export ATP-binding/permease protein MacB (EC 3.6.3-)**	1036.871	426.512	0.000
**Macrolide-specific efflux protein MacA**	210.917	668.432	0.000
**Multi antimicrobial extrusion protein (Na(+)/drug antiporter-). MATE family of MDR efflux pumps**	8815.673	3125.98	0.000
Multidrug efflux pump component MtrF	19.153	2.949	0.000
**Multidrug efflux RND membrane fusion protein MexC**	4.113	1.517	0.022
**Multidrug efflux RND transporter MexD**	19.084	6.185	0.002
Multidrug resistance efflux pump PmrA	20.989	3.974	0.000
Multidrug-efflux transporter. major facilitator superfamily (MFS) (TC 2.A.1)	14.734	47.616	0.000
Transcription regulator of multidrug efflux pump operon. TetR (AcrR) family	22.021	4.136	0.000
Transcription repressor of multidrug efflux pump acrAB operon. TetR(AcrR) family	4.972	24.051	0.001
Multiple antibiotic resistance protein MarA	0.307	3.607	0.003
Level3- Polymyxin_Synthetase_Gene_Cluster_in_Bacillus			
Polymyxin synthetase PmxA	1.953	0.255	0.029
Level 3- Resistance_to_fluoroquinolones			
Efflux pump Lde	25.941	69.768	0.024

**Topoisomerase IV subunit B (EC 5.99.1-)**	1796.567	0	0.000
Level 3- Resistance_to_Vancomycin			
Protein VanZ	1.472	0.085	0.027
**Sensor histidine kinase VanS (EC 2.7.3)**	847.058	136.855	0.000
**Vancomycin B-type resistance protein VanW**	882.44	147.837	0.000
Vancomycin B-type resistance protein VanX	65.047	29.341	0.000
Vancomycin resistance protein VanH	11.766	4.74	0.015
**Vancomycin response regulator VanR**	1340.459	856.222	0.005
Vancomycin Teicoplanin A-type resistance protein VanA	44.3	3.047	0.000
Level 3- Streptococcus_pneumoniae_Vancomycin_Tolerance_Locus			
ABC transporter. ATP-binding protein Vex2	84.104	8.606	0.000
Level 3- Streptothricin_resistance			
**Streptothricin acetyltransferase. Streptomyces lavendulae type**	149.21	248.089	0.001
Level 3- The_mdtABCD_multidrug_resistance_cluster			
**Multidrug transporter MdtB**	16.731	79.835	0.005
**Multidrug transporter MdtC**	15.583	69.334	0.002
**Multidrug transporter MdtD**	7.577	32.849	0.003
**Probable RND efflux membrane fusion protein**	709.051	1590.562	0.000
**Response regulator BaeR**	53.501	23.835	0.002
Sensory histidine kinase BaeS	16.449	35.314	0.019
